# Voluntary & Community Organisations: Not the Third but the First Sector of Integrated Care?

**DOI:** 10.5334/ijic.10183

**Published:** 2025-09-19

**Authors:** Robin Miller, Michelle Nelson, Ivette Fullerton, Felix Gradinger, James Rees, Marianne Saragosa

**Affiliations:** 1 University of Birmingham, UK; 2 University of Toronto, CA; 3 Cihuapactli Collective, US; 4 University of Plymouth, UK; 5 University of Wolverhampton, UK; 6 Science of Care Institute, Sinai Health, CA

**Keywords:** Voluntary and Community Sector, Third Sector, charity, integrated care

When Mari, a person with diabetes and mental health challenges experiencing homelessness needed care, the hospital could treat their immediate health challenges but couldn’t address why they kept returning. It was a small community organization, with deep neighbourhood knowledge, flexible funding, and trusted relationships—that provided the wraparound support that finally broke the cycle. This story, repeated countless times across health systems worldwide, raises a fundamental question: Why do we refer to the voluntary and community sector (VCS) as the “third sector” when it’s often the first to respond, innovate, and reach those most in need?

Over the past decade, integrated care has evolved beyond coordinating medical services to embrace truly holistic, person-centred approaches that address social determinants of health. This shift has highlighted the essential role of voluntary and community organizations, yet their contributions remain undervalued, underfunded, and poorly understood within health systems. This special issue challenges that positioning, presenting evidence that the VCS may be better positioned than traditional health services to deliver the values and outcomes that integrated care promises.

The voluntary and community sector encompasses an extraordinary diversity of organizations, from volunteer-run food banks serving a single neighbourhood to global networks addressing systemic inequities. What unites these organizations across their varied forms is not structure but purpose: they prioritize mission over profit, community empowerment over institutional power, and social justice over efficiency metrics. Common values include social justice and equity, community empowerment and participation, inclusivity and diversity, as well as collaborative approaches to addressing societal challenges. This diversity enables VCS organizations to adapt rapidly to emerging needs, innovate beyond regulatory constraints, and develop culturally responsive approaches that larger institutions cannot match. Yet this same flexibility creates perception problems. To public sector professionals accustomed to standardized processes and clear hierarchies, the VCS can appear “fragile, volatile, messy and confusing,” despite representing some of society’s most enduring institutions (for further history and background to the VCS, please see Rees & Mullins [1] and Carmichael & Elson [2]).

While there are dedicated journals which focus on the VCS (e.g. Voluntas, NVSQ, and Voluntary Sector Review), which suggests a certain degree of researcher activity, the research funding landscape reveals a troubling paradox: the organizations best positioned to address health inequities often receive the least support to demonstrate their impact. While medical technologies and clinical interventions attract millions in research funding, VCS initiatives frequently struggle to secure even modest grants for evaluation. Many VCS organizations lack the administrative infrastructure that universities and large health systems use to navigate complex grant applications. Research funding panels, dominated by academics with limited VCS experience, often view community organizations as insufficiently “robust” for rigorous study and subsequently invest in ‘what they know.’ Meanwhile, a wealth of innovation and learning takes place within the sector through grassroots evaluation, policy briefs, and quality improvement efforts that often remain unpublished in academic venues. The result is a dangerous “evidence gap” where the approaches most needed for integrated care—community-centred, relationship-based, culturally responsive, cross-boundary, inter-sectorial —appear to lack scientific support, not because they don’t work, but because they haven’t been studied with traditional research methods.

Such disparities are as true of integrated care as in other fields. Despite the VCS being “situated in the communities they serve, having a deep understanding of members’ needs, and being in a trusted position to help” [3 (p3)] they can struggle for their contribution to be properly recognised, endorsed, and funded. This includes the International Journal of Integrated Care, where the number of articles falls disproportionately below the level of activity and impacts of the VCS on integrated care. This Special Issue is a response to this gap and has been edited by a team with lived, practice and research experience of the VCS. Submissions, spanning Europe, North America, East Asia, and Australasia, tell a different story about the role of VCS in integrated care. We received over twenty articles with a higher proportion of perspectives and practice innovations (Integrated Care Cases) relative to research articles than is usual, likely reflecting the bias in access to funding outlined above as well as the complexity and diversity of integrated care support provided by the sector. Rather than bit players in integrated care, VCS organizations emerge as sophisticated integrators who excel at the very challenges that stump traditional health systems.

The breadth of populations supported within the special issue is considerable, with particular focus on responding to the needs of those who are often excluded from society and therefore face systemic barriers to accessing appropriate support. This includes those with issues related to substance misuse, homelessness, abuse, and severe mental health difficulties, alongside young people transitioning into adult services, older people with dementia, and people living with cancer. These are precisely the groups that slip through the cracks of conventional care coordination. The articles outline how the VCS has developed innovative responses to improve wellbeing, with VCS organizations not just filling gaps but creating entirely new pathways that work for people whom traditional systems have failed.

As one of our editors reflected, “There’s complexity of populations and complexity of pathways… but they’re comfortable with that complexity.” This comfort with complexity—rather than attempts to standardize it away—may be exactly what integrated care needs. The articles document how VCS organisations facilitate vertically and horizontally integrated care through three key functions: navigation and access, where they serve as bridges helping people understand and access complex health systems while advocating for their needs; care coordination that is relationship-based and person-directed, adapting to individual circumstances rather than institutional protocols; and providing platforms for community voice, enabling communities to identify their own needs, mobilize their own assets, and advocate for systemic changes.

In many respects, the special issue suggests that the VCS sector is better able to demonstrate the values of integrated care than mainstream health and care services. Despite these contributions, there were significant challenges to VCS organisations being able to fully contribute to integrated care partnerships. These included hospital providers dominating through their size, status, and professional expertise; funders curtailing VCS autonomy and distinctiveness through tightly drawn contracts and metrics; unjustified concerns about their professionalism and delivery expertise; and inadequate and unpredictable funding. Professional hierarchies privilege medical and clinical expertise over community knowledge and lived experience, while short-term, restrictive contracts force VCS organizations to demonstrate outcomes using metrics designed for clinical interventions rather than community development. As an editor put it, “even within the best teams, VCS partners often still feel like they’ve got the kids’ table dragged up to the adult dinner table.”

The special issue also contains practical approaches to addressing these obstacles through changing the dynamics between the VCS and public sectors. Successful integrated care partnerships demonstrate several key characteristics: inclusive governance where VCS organizations have genuine decision-making power, including board positions, budget authority, and leadership opportunities; capacity investment that builds VCS capability for research, evaluation, and system leadership rather than expecting them to participate on existing terms; flexible funding where contracts reflect the actual work requested, including relationship-building, community development, and advocacy; and mutual education, where health professionals learn about community organizing and asset-based development while VCS leaders gain insight into clinical pathways and health system constraints. As one editor noted, “you shouldn’t have to make a financial sacrifice to do something that society needs.”

The richness of the special issue suggests that there is much more to be learnt from researching the role and realities of the VCS within integrated care. This relates not only to how their contribution can be better recognised and supported, but also to how integrated care in general can be organised and led. Their ability to develop accessible, person-centred, and asset-based approaches with populations who are often marginalised and discriminated against within society should inspire, inform and challenge other health and care professionals and delivery organisations. This learning potential also relates to research, with the VCS’s ability to engage and co-produce with communities, bringing skills and capacity which will help integrated care researchers to better focus on what matters to people and recognise the practical implications of their findings.

Realising this potential will require researchers to collaborate as equals, build in the resources required by the VCS within funding bids, and develop inclusive ways of working. As one of our editors reflected, “Language is always one of the barriers… There needs to be a bridge between research, policy, and community organisations.” Perhaps it’s time to stop asking how voluntary and community organizations can better fit into integrated care systems and start asking how integrated care systems can better learn from VCS approaches. The evidence in this special issue suggests that many VCS organizations are already delivering the kind of person-centered, community-responsive, equity-focused care that integrated care aspires to achieve. The question isn’t how VCS organizations sit at the table—it’s whether we’re ready to acknowledge that they may have been setting the table all along.
